# Association between macular edema area on optical coherence tomography and Amsler grid sensitivity in diabetic macular edema: a cross-sectional study

**DOI:** 10.1186/s12886-026-05298-3

**Published:** 2026-09-05

**Authors:** Sophie Wolf, Georg Michelson

**Affiliations:** 1 Department of Ophthalmology, Universitätsklinikum Erlangen and Friedrich-Alexander-Universität Erlangen-Nürnberg (FAU), Schwabachanlage 6, 91054 Erlangen, Germany; 2 Talkingeyes & More GmbH, Henkestraße 91, 91052 Erlangen, Germany

**Keywords:** Diabetic macular edema, Screening, Amsler grid, Macular edema area, Optical coherence tomography

## Abstract

**Background:**

Early detection of diabetic macular edema (DME) is essential to prevent vision loss through timely treatment. Optical coherence tomography (OCT) is the diagnostic gold standard but is not suitable for home monitoring. The Amsler grid represents a simple and inexpensive potential screening tool. This study aimed to determine the sensitivity of the Amsler grid for detecting DME and to assess its association with the size of foveal edema.

**Methods:**

In this cross-sectional study, 1,444 eyes of 722 diabetic patients were examined using Spectralis OCT and the Amsler grid, and 90 eyes with DME were identified. A sensitivity analysis restricted to one eye per participant and excluding eyes with concomitant macular pathologies included 34 eyes with intrafoveal DME, in which the area of foveal edema was quantified.

**Results:**

For very small DME (area ≤ 0.01 mm²), the sensitivity of the Amsler grid was 20.0% (1/5 eyes). For edema areas > 0.01 mm², the sensitivity increased to 62.1% (18/29 eyes), and for areas > 0.03 mm², it increased to 69.6% (16/23 eyes).

**Conclusion:**

The Amsler grid demonstrated limited sensitivity for detecting DME, particularly for small edema areas (≤ 0.01 mm²). While it may serve as an adjunctive tool for home monitoring in diabetic patients, it cannot replace OCT-based examinations at recommended follow-up intervals.

## Background

Diabetic macular edema (DME) is the main cause of vision loss in diabetic retinopathy (DR) and represents a major cause of visual impairment among working-age adults in developed countries. Driven by increasing life expectancy and rising rates of obesity, the global prevalence of diabetes mellitus continues to increase [1]. According to the most recent International Diabetes Federation (IDF) Diabetes Atlas, approximately 589 million adults worldwide (11.1% of the global adult population) were living with diabetes in 2024, and this number is projected to increase to 853 million (13.0%) by 2050 [2]. A meta-analysis published in 2022 estimated that the prevalence of DME among individuals with diabetes was approximately 5.5% [3]. Long-term studies report a 10-year incidence of DME of around 20% in patients diagnosed with diabetes before the age of 30 and nearly 40% in those diagnosed after the age of 30 [4]. 

Timely treatment can improve or stabilise visual acuity [5]. However, visual impairment often occurs only when retinal damage has already progressed considerably, at which point the chances of successful treatment may be reduced. Therefore, early detection of DME is essential [6]. For this reason, regular ophthalmological examinations are recommended for individuals with diabetes. Depending on the individual risk profile, ophthalmological screening of the ocular fundus is generally recommended every 1–2 years in patients without signs of DR. If retinal damage is already present, shorter follow-up intervals are advised [7]. 

Nevertheless, studies indicate that approximately 30–50% of patients with diabetes do not undergo ophthalmological screening at the recommended intervals [8]. The cost of current diagnostic methods and the limited availability of ophthalmologists represent important barriers to achieving the recommended screening coverage [9]. One approach to address this challenge is the use of telemedicine with remote grading. Telemedicine screening approaches have demonstrated their effectiveness and usefulness, especially during the coronavirus disease 2019 (COVID-19) pandemic [10]. However, imaging modalities such as optical coherence tomography (OCT) require specialised equipment and expert evaluation, are relatively costly, and are therefore unsuitable for home monitoring.

In 1947, the Swiss ophthalmologist Marc Amsler developed a printed grid for detecting age-related macular degeneration (AMD), which he termed the Amsler grid. This test is now widely used as a simple and inexpensive tool for monitoring macular disease at home [11]. The Amsler test allows the detection and monitoring of metamorphopsia and scotoma in the central visual field and is primarily used for the early detection and monitoring of neovascular AMD. However, it may also be applied in other retinal diseases, including central serous chorioretinopathy (CSCR), epiretinal membrane, acute macular neuroretinopathy, and cystoid macular edema [12]. Although the Amsler grid has been used in clinical practice for many years, reliable data on its diagnostic performance for detecting macular diseases other than AMD remain limited.

However, the usefulness of the Amsler grid for detecting DME has not been sufficiently investigated, particularly in relation to edema size and test sensitivity. A simple and inexpensive self-monitoring tool could improve the early detection of DME, especially in patients who do not adhere to recommended screening intervals. Therefore, the aim of this study was to evaluate the diagnostic performance of the Amsler grid as a potential screening test for DME. Specifically, we analysed the relationship between the area of DME in the foveal region and the sensitivity of the Amsler test. In addition, we also assessed the sensitivity and specificity of the Amsler grid for selected retinal pathologies.

## Methods

### Study population

After approval was obtained from the Ethics Committee of the Friedrich-Alexander-Universität Erlangen-Nürnberg, the study was conducted between May 2019 and March 2021 and included 722 subjects with diabetes mellitus. Participants were randomly recruited from nine diabetes care practices in Germany. The inclusion criteria were a diagnosis of diabetes mellitus and written informed consent to participate in the study. The exclusion criteria were incomplete datasets and insufficient image quality of the corresponding examinations.

As part of the study protocol, participants were asked a standardized set of questions, and their responses were documented. The questionnaire covered cardiovascular and cerebrovascular history, diabetes-related complications, cardiovascular risk factors, smoking history, anthropometric characteristics, refractive status, and relevant ocular history, including elevated intraocular pressure, age-related macular degeneration, previous ocular surgery, and previous laser treatment. These data were collected for the overall study protocol but were not systematically analyzed in the present study, as they were beyond the scope of the study objective.

Among the participants, 45.7% were female. The mean age was 60.49 ± 16.1 years, the mean duration of diabetes was 19.13 ± 10.84 years, and the mean glycated haemoglobin (HbA1c) level was 7.2 ± 1.1%, based on medical history.

### Image acquisition and Amsler grid testing

All examinations were performed by a trained technician in diabetes care practices. Ocular fundus imaging was conducted using a Spectralis OCT device and a multispectral camera (Heidelberg Engineering). For each eye, five images were obtained: a macula-centred cross-section in the vertical and horizontal orientations, a MultiColour (MC) image centred on the macula and the optic disc, and a ring scan of the optic disc to determine retinal nerve fibre layer thickness. Both eyes of each participant were examined.

In addition, the Amsler grid test was performed using a standard medical Amsler grid displayed on a tablet with black lines on a white background. The grid measured 10 × 10 cm and consisted of 400 small squares with a grid spacing of 0.5 cm. Each eye was tested separately at a reading distance of approximately 30–40 cm. Testing was performed under regular practice lighting conditions, and the participants’ own near-vision correction was used when available. The participants were instructed to fixate on the central point and to circle any perceived distortions of the grid lines using a touch pen. If the participant marked any area on the grid, the test result was considered positive.

### Telemedical assessment

The ocular fundus images and the results of the Amsler grid test were uploaded and stored in the cloud-based patient file MedStage^®^ (from Talkingeyes & More GmbH). This platform is an electronic medical record system for ophthalmic diseases and was certified as a class IIa medical device by TÜV Rheinland in October 2018. It enables structured storage of clinical data and images as well as telemedical diagnostic assessment [13]. 

Subsequently, a telemedical ophthalmological evaluation was performed by an experienced ophthalmologist (GM). The central fundus was assessed for retinal vascular tortuosity, thickened veins, microaneurysms, arteriovenous crossings, hard exudates, retinal haemorrhages, and arterial constrictions. In addition, the degree of hypertensive and diabetic retinopathy was determined, and pathologies of the optic disc and macula were documented within the MedStage^®^ platform.

### Quantification of retinal parameters

Retinal and choroidal thickness in the central foveal region as well as the area of DME were measured manually using the Heidelberg Eye Explorer software (HEYEX) from Heidelberg Engineering. Macula-centred cross-sectional OCT images were used for the measurements. Retinal thickness was measured at the deepest point of the central fovea.

For the assessment of DME, the OCT cross-sectional image with the largest visible extent of the intraretinal fluid was selected. The distinction between intrafoveolar and extrafoveolar DME was based on the anatomical location of the fluid in relation to the foveal depression on the OCT image. An eye could have intrafoveolar DME, extrafoveolar DME, or both. On the OCT images, the intraretinal fluid appeared as a hyporeflective, sharply demarcated area. This area was manually outlined, and the enclosed area was calculated automatically by the software. If multiple fluid compartments were present, their areas were summed to obtain the total cross-sectional area. The resulting cross-sectional area is referred to throughout this manuscript as the “DME area”. The OCT images were evaluated independently of the Amsler grid results.

All findings and measurement results were exported from the MedStage^®^ platform into a Microsoft Excel spreadsheet for statistical analysis.

### Analysis populations

For the evaluation of the diagnostic performance of the Amsler grid for different retinal pathologies (Table 2), all eyes with evaluable OCT and Amsler grid results were included in the analysis.

For analyses evaluating the relationship between intrafoveolar DME area and Amsler grid sensitivity (Tables 3 and 4), eyes with exclusively extrafoveolar DME were excluded. To minimize potential confounding, eyes with concomitant macular diseases that could influence Amsler grid performance, such as age-related macular degeneration, epiretinal membrane, vitreomacular traction, retinoschisis, or other clinically relevant macular pathologies, were also excluded. To avoid inter-eye correlation, only one eye per patient was included. In patients with bilateral eligible eyes, the right eye was selected for analysis.

### Statistical analysis

Statistical analyses were performed using IBM SPSS Statistics (version 27.0).

Linear regression analysis was used to evaluate the correlation between central retinal thickness and the area of DME. A p-value < 0.05 was considered statistically significant. Exact 95% confidence intervals for sensitivity were calculated using the Clopper–Pearson method (OpenEpi, Version 3).

## Results

### OCT findings

Table 1 summarises the prevalence of anatomical abnormalities detected in the OCT images. Various retinal pathologies were identified, including DME, which was classified as intrafoveolar or extrafoveolar. In addition, vitreoretinal interface abnormalities such as epiretinal membrane and vitreomacular traction were observed. Other findings included AMD and associated features such as drusen, retinal pigment epithelium irregularities, photoreceptor abnormalities, and pigment epithelial detachment. Additional retinal conditions identified in the study population included adult vitelliform macular dystrophy, chorioretinal atrophy, fibro-disciform maculopathy, and retinoschisis.

**Fig. 1:**
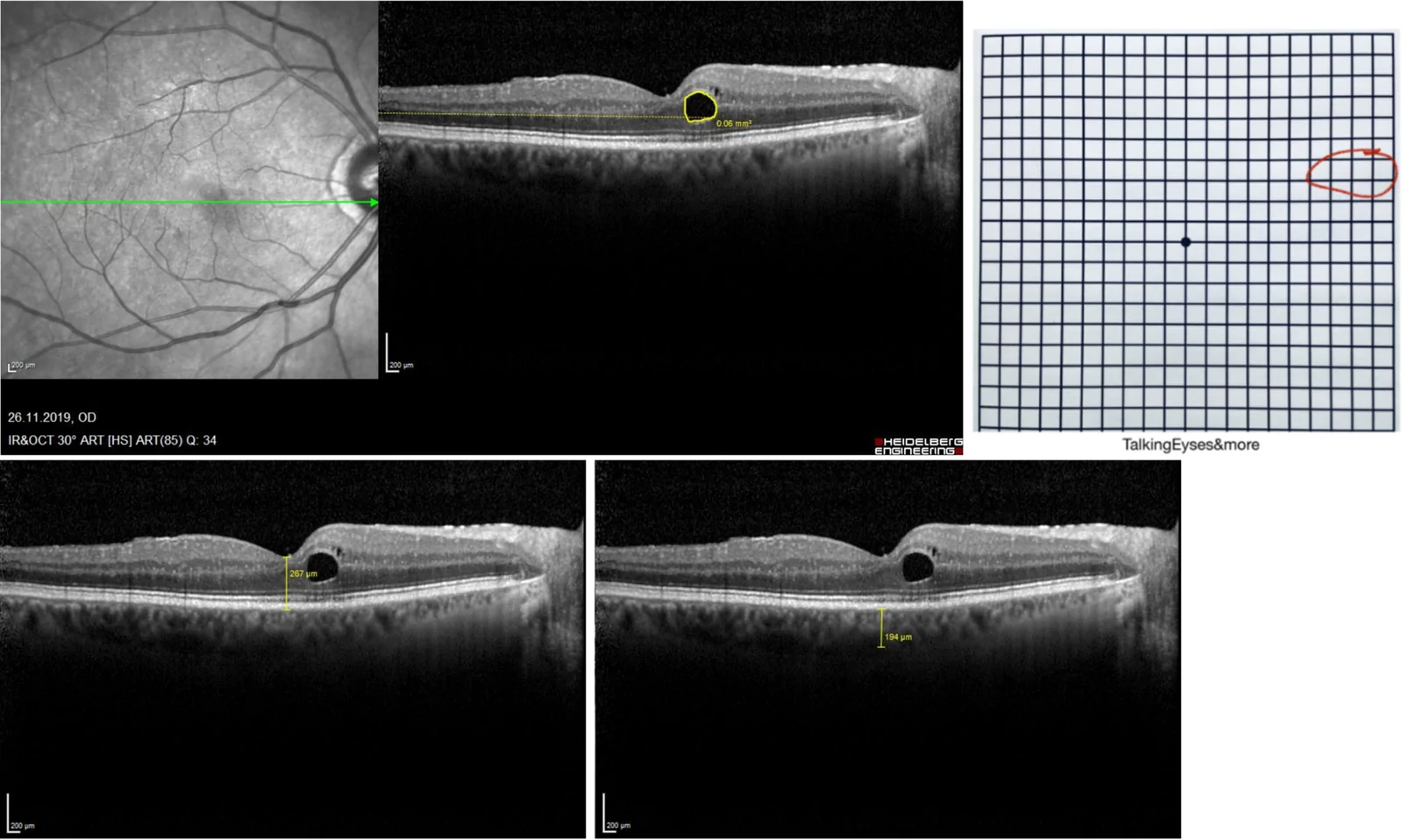
Example images showing measurements of retinal and choroidal thickness and DME area using the HEYEX software, along with the corresponding Amsler grid

Using OCT images, central retinal thickness, choroidal thickness, and the area of DME were measured with the HEYEX software. The mean retinal thickness was 236.5 ± 39 μm (*N* = 1,430 eyes), and the mean choroidal thickness was 205.9 ± 48.7 μm (*N* = 1,431 eyes). The mean DME area was 0.07 ± 0.09 mm² (*N* = 88 eyes). Figure 1 shows an example of DME with measurements of retinal thickness, choroidal thickness, and diabetic macular edema area (DME area), along with the corresponding Amsler grid result.

Due to missing or non-evaluable measurements, the number of eyes available for individual analyses varied.

### Correlation between retinal thickness and DME area

**Fig. 2:**
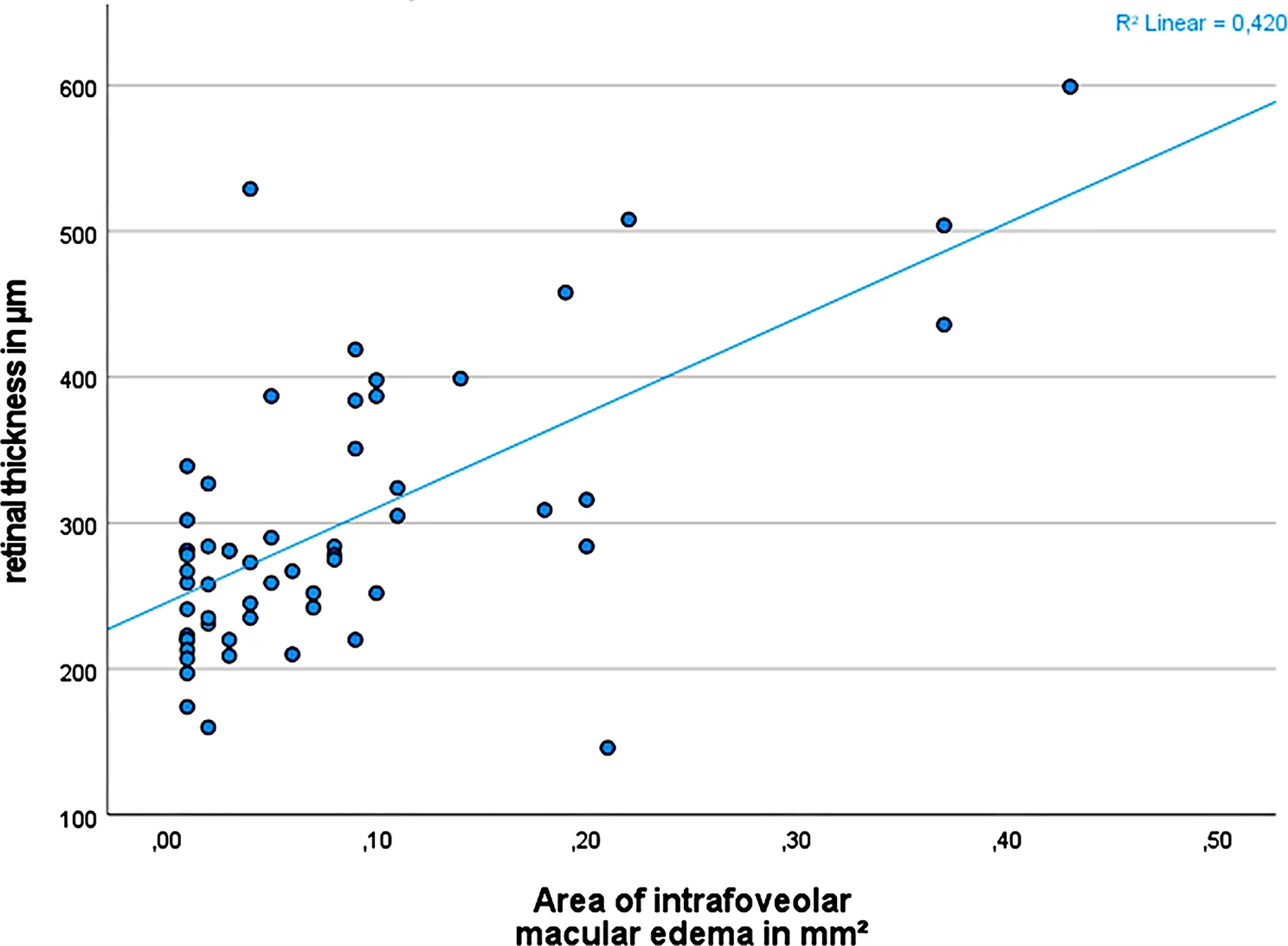
Scatter plot of retinal thickness vs. intrafoveolar DME area

Linear regression analysis revealed a significant positive correlation between central retinal thickness and the area of intrafoveolar DME (Pearson *r* = 0.65, R² = 0.42, *p* < 0.001; Fig. 2), indicating that retinal thickness increased with increasing DME area.

### Amsler grid findings

A total of 1,397 Amsler grid test results were available for analysis, of which 5.3% (*n* = 76) were positive. In 6.6% (*n* = 5) of the positive tests, no retinal pathology was detected.

The diagnostic performance of the Amsler grid for detecting different retinal pathologies was evaluated by calculating sensitivities and specificities (Table 2). Specificity was consistently high across all conditions (94.6–96.5%), whereas sensitivity varied widely (0.0–80.0%). For retinal pathologies with small numbers of affected eyes, the corresponding 95% confidence intervals were wide, indicating limited precision of the sensitivity estimates.

The highest sensitivity was observed for fibro-disciform maculopathy (80%), followed by AMD (77.8%), lifted foveal depression (57.7%), and intrafoveolar DME (51.9%).

### Effect of DME area on Amsler grid sensitivity

**Fig. 3:**
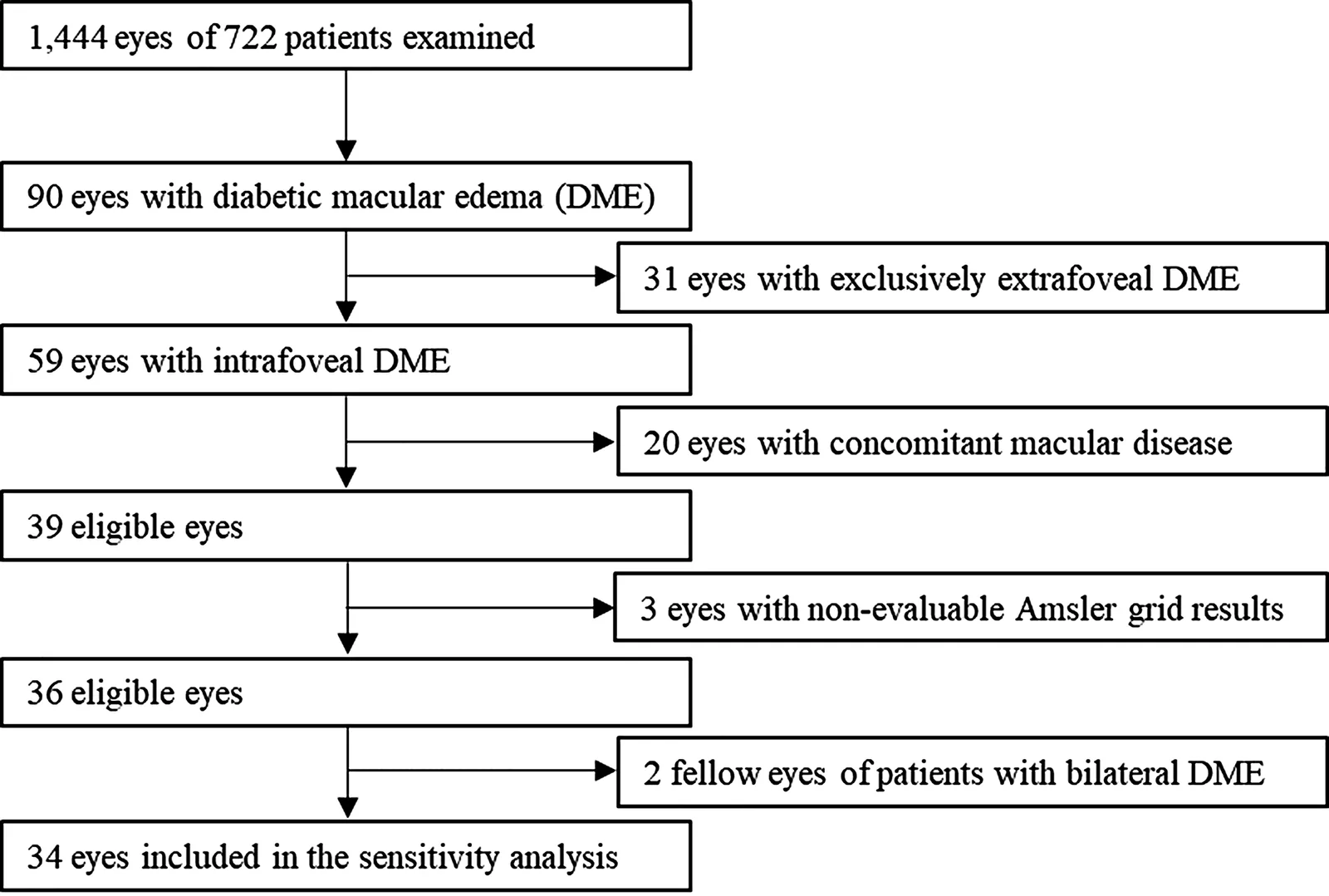
Flow diagram of eye selection for the intrafoveolar DME sensitivity analysis

The selection of eyes included in the sensitivity analysis is shown in Fig. 3. Of the 90 eyes with DME, 31 had exclusively extrafoveal edema and were therefore excluded. After excluding eyes with concomitant macular disease, eyes with non-evaluable Amsler grid results, and fellow eyes from patients with bilateral eligible DME, 34 eyes remained for the final sensitivity analysis.

The influence of intrafoveolar DME area on Amsler grid sensitivity was analysed by grouping eyes according to edema area (Table 3).

A more detailed analysis across individual DME area is presented in Table 4. Sensitivity varied considerably between individual edema area and showed no consistent pattern. For very small edema (area ≤ 0.01 mm²), sensitivity was low at 20.0% (1/5). Although higher sensitivities were observed in some subgroups, these estimates were based on small sample sizes and were accompanied by wide 95% confidence intervals.

Cumulative analysis showed that sensitivity increased to 62.1% (18/29) for DME areas greater than 0.01 mm² and to 69.6% (16/23) for areas greater than 0.03 mm². The highest cumulative sensitivity was observed for DME areas greater than 0.09 mm² (80.0%, 8/10). Overall, sensitivity appeared to be higher for larger edema areas, although no consistent or linear relationship was observed.

**Table 1:** Prevalence of OCT findings

OCT findings	Prevalence (*N* = 1,437)	
	*n*	%
Diabetic macular edema (overall)	90	6.3
-intrafoveolar	59	4.1
-extrafoveolar	57	4.0
Retinal pigment epithelium irregularities	47	3.3
Photoreceptor abnormalities	27	1.9
Drusen	56	3.9
Epiretinal membrane	56	3.9
Lifted foveal depression	28	1.9
Pigment epithelial detachment	5	0.3
Retinoschisis	5	0.3
Age-related macular degeneration	11	0.8
Vitreomacular traction	16	1.1
Hyperreflective foci	27	1.9
Adult vitelliform macular dystrophy	7	0.5
Chorioretinal atrophy	7	0.5
Fibro-disciform maculopathy	5	0.3
Retinal haemorrhage	8	0.6

**Table 2:** Sensitivity, specificity and 95% confidence intervals of the Amsler grid for detecting retinal pathologies based on OCT findings

OCT findings	*n*	Sensitivity (%)	95% CI	Specificity (%)	95% CI
Diabetic macular edema - intrafoveolar	54	51.9	37.8–65.7	96.5	95.4–97.4
Diabetic macular edema - extrafoveolar	50	38.0	24.7–52.8	95.8	94.6–96.8
Retinal pigment epithelium irregularities	44	31.8	18.6–47.6	95.5	94.2–96.5
Photoreceptor abnormalities	26	46.2	26.6–66.6	95.4	94.2–96.5
Drusen	54	14.8	6.6–27.1	95.0	93.7–96.1
Epiretinal membrane	56	19.6	10.2–32.4	95.2	93.9–96.3
Lifted foveal depression	26	57.7	36.9–76.6	95.6	94.4–96.6
Pigment epithelial detachment	5	40.0	5.3–85.3	94.7	93.4–95.9
Retinoschisis	5	20.0	0.5–71.6	94.7	93.4–95.8
Age-related macular degeneration	9	77.8	40.0–97.2	95.1	93.8–96.2
Vitreomacular traction	16	18.8	4.0–45.6	94.8	93.5–95.9
Hyperreflective foci	27	25.9	11.1–46.3	95.0	93.7–96.1
Adult vitelliform macular dystrophy	7	14.3	0.4–57.9	94.7	93.4–95.8
Chorioretinal atrophy	4	25.0	0.6–80.6	94.7	93.4–95.8
Fibro-disciform maculopathy	5	80.0	28.4–99.5	94.9	93.6–96.0
Retinal haemorrhage	8	0.0	0.0–36.9	94.6	93.3–95.7

**Table 3:** Sensitivity of the Amsler grid by intrafoveolar DME area (grouped) (*N* = 34)

DME area (mm²)	Positive, *n*	Negative, *n*	Sensitivity (%)	95% CI
0.01–0.05	5	10	33.3	11.8–61.6
0.06–0.10	9	3	75.0	42.8–94.5
0.11–0.15	2	1	66.7	9.4–99.2
0.16–0.20	2	0	100.0	15.8 − 100.0
> 0.21	1	1	50.0	1.3 − 98.7
**Total (overall)**	19	15	55.9	37.9 − 72.8

**Table 4:** Sensitivity of the Amsler grid by intrafoveolar DME area: individual and cumulative analysis (*N* = 34)

DME area (mm²)	Positive, *n*	Negative, *n*	Sensitivity (%)	cumulative DME area (mm²)	Sensitivity (*n*/*N*)	Sensitivity (%)
0.01	1	4	20.0			
0.02	2	1	66.7	> 0.01	18/29	62.1
0.03	0	3	0.0	> 0.02	16/26	61.5
0.04	1	0	100.0	> 0.03	16/23	69.6
0.05	1	2	33.3	> 0.04	15/22	68.2
0.06	2	0	100.0	> 0.05	14/19	73.7
0.07	1	1	50.0	> 0.06	12/17	70.6
0.08	2	1	66.7	> 0.07	11/15	73.3
0.09	1	1	50.0	> 0.08	9/12	75.0
0.10	3	0	100.0	> 0.09	8/10	80.0
0.11	2	0	100.0	> 0.10	5/7	71.4
0.14	0	1	0.0	> 0.11	3/5	60.0
0.19	1	0	100.0	> 0.14	3/4	75.0
0.20	1	0	100.0	> 0.19	2/3	66.7
0.37	0	1	0.0	> 0.20	1/2	50.0
0.43	1	0	100.0	> 0.37	1/1	100.0
Total	19	15	55.9			

## Discussion

### Assessment of DME extent by OCT

DME is defined as central retinal thickening, possibly with lipid exudates. Different forms are distinguished, such as focal, diffuse, and tractional DME, as well as ischaemic maculopathy and mixed forms. The classification depends on the findings and the presence of other pathologies [5]. 

The term “clinically significant macular edema” was historically relevant but now plays a minor role. Today, differentiation into DME with and without foveal involvement is standard and is typically based on OCT findings. This classification influences follow-up intervals and treatment decisions [7]. For this reason, we applied this classification in our study. In our analysis, we focused on edema with foveal involvement (intrafoveolar), as edema without foveal involvement has a limited impact on central vision and rarely leads to metamorphopsia.

Basic diagnostic methods for DME include biomicroscopic fundus examination during mydriasis [5]. Fundus photography and fluorescein angiography can also be used to assess the presence of DME. However, these methods have been largely superseded by OCT, which allows precise localisation and quantification of edema and is highly reproducible [14]. OCT can detect subclinical macular edema before functional impairment occurs and can therefore be used for early diagnosis. Furthermore, it is essential for therapy monitoring [5, 15]. 

Determination of central retinal thickness is relevant both for indication and for assessing the effectiveness of treatment [6]. Central subfield thickness (CST) is used as a quantifiable biomarker for DME diagnosis. The diagnostic threshold is commonly defined as the mean machine-specific OCT-measured thickness in normative eyes of an individual with diabetes without macular edema plus two standard deviations. In the Diabetic Retinopathy Clinical Research Network (DRCR.net) study, which also used the Heidelberg Spectralis OCT machine, the CST threshold was set at 320 μm in male patients and 305 μm in female patients [16]. However, the threshold is also frequently set at 300 μm in general [17–20]. CST is dependent on many factors such as age, sex and the presence of other retinal pathologies and is therefore not an ideal standalone parameter for diagnosing DME. A 2015 Cochrane review also reported that CST measurement is not sufficiently accurate to diagnose DME [21]. The main feature of CME is exudative fluid accumulation in the macula, which is not present in a healthy retina. Therefore, direct assessment of intraretinal fluid may be more sensitive and specific than measurement of central retinal thickness alone. You et al. hypothesised that measuring macular fluid volume on dense volumetric OCT scans may provide higher diagnostic accuracy than central subfield thickness for DME screening [16]. 

Rather than using CST or volumetric OCT parameters, we quantified the maximum cross-sectional area of intraretinal fluid because our primary objective was to investigate whether the morphologically visible extent of intraretinal fluid was associated with the subjective perception of metamorphopsia. A significant correlation between central retinal thickness and the area of intrafoveal DME was observed.

### Diagnostic performance of the Amsler grid for DME

The Amsler test has been an important tool for the detection and monitoring of macular diseases for many years. However, several known limitations should be considered when evaluating the test. These include „its non-interactive nature, missing fixation control, need for reasonable reading vision to discern the lines, low sensitivity due to a suprathreshold stimulus, its poor performance due to the ‘crowding effect’, and limited awareness of visual field defects until the scotoma is significantly large in size due to ‘filling-in phenomenon’” [22]. To minimise these limitations, many modifications have been developed in recent years to increase the sensitivity of the test, such as the Threshold Amsler chart or the Sine Amsler chart [12]. Computerised methods for home monitoring of metamorphopsia, including the Preferential Hyperacuity Perimeter and the three-dimensional computer-automated threshold Amsler grid, have also been developed [11]. Some of the newer methods show higher accuracy in detecting scotomas and metamorphopsias. Programmes have also been developed that make it possible to quantify metamorphopsia. One example is the “AMD - A Metamorphopsia Detector”, which is based on the Amsler test but calculates an overall metamorphopsia index (MI) by analysing the amplitude, position, and extension of the distorted perceived lines. Claessens and Schuster investigated the correlation of this MI with central retinal thickness (CRT), which was high in eyes with DME (rho = 0.88) [17]. Based on the software AMD - A Metamorphopsia Detector, an app called “MacuFix” has been developed that has the potential to improve vision-related quality of life in patients with metamorphopsia due to various retinal diseases [23]. 

The Amsler grid was presented digitally on a tablet in our study. Although we assume that our findings may be transferable to the traditional printed Amsler grid and modified versions of the test, this has not been specifically validated and should therefore be interpreted with caution.

In our overall study population, 51.9% of eyes with intrafoveolar DME were correctly identified using the Amsler grid, whereas specificity was high at 96.5%. These values are higher than those reported in previous studies. For example, Brown et al. reported a sensitivity of 37.5% and a specificity of 73.8% for detecting clinically significant macular edema in 2000 [24]. However, when all DME cases (including those without foveal involvement) were considered in our cohort, sensitivity decreased to 39.0%, while specificity remained high at 96.7%.

In the selected study population used for the DME area analysis, sensitivity varied according to DME area. For very small DME areas (≤ 0.01 mm²), sensitivity was low at 20.0%. For DME areas greater than 0.01 mm², sensitivity increased to 62.1% and to 69.6% for areas greater than 0.03 mm². However, no consistent increase in sensitivity with increasing edema area was observed; thus, our initial hypothesis could not be confirmed.

Larger DME areas were relatively uncommon in our cohort. Only seven eyes had a DME area greater than 0.10 mm², of which five showed a positive Amsler grid result, indicating that 28.6% did not report metamorphopsia. One possible explanation is reduced best-corrected visual acuity (BCVA) in advanced disease, which may limit the perception of visual distortions. However, BCVA was not assessed in this study.

The wide confidence intervals observed in several DME area categories reflect the small number of eyes in these subgroups and limit the precision of the sensitivity estimates.

Taken together, these findings indicate that the Amsler grid may have limited sensitivity for detecting small DME and is therefore not suitable as a standalone screening tool for early-stage disease.

### Diagnostic performance of the Amsler grid for other retinal pathologies

The Amsler grid has been used since 1947 to detect and monitor metamorphopsia associated with macular disease and is primarily applied as a home monitoring tool for neovascular AMD. Despite its long-standing use in clinical practice, data on its sensitivity for detecting maculopathies other than AMD remain limited.

Klatt et al. evaluated the sensitivity of the Amsler test in six macular diseases and reported sensitivities of 87% for macular holes, 100% for epiretinal membrane, 73% for central serous chorioretinopathy, 100% for intermediate AMD, 94% for classic choroidal neovascularisation, and 71% for occult choroidal neovascularisation [25]. 

A review by Tripathy and Salini (2020) described a wide range of conditions in which the Amsler grid may be used, including neovascular AMD, central serous chorioretinopathy, epiretinal membrane and other vitreoretinal interface disorders, acute macular neuroretinopathy, and cystoid macular edema of various etiologies. In addition, neurological and systemic conditions such as non-arteritic anterior ischaemic optic neuropathy, pituitary tumours, and hydroxychloroquine retinopathy were mentioned. However, quantitative data on sensitivity were not provided [12]. 

We considered the sensitivities and specificities for different retinal pathologies individually and found that the Amsler test was most sensitive for the detection of fibro-disciform maculopathy (80%), AMD (77.8%), lifted foveal depression (57.7%) and intrafoveolar DME (51.9%). However, the false-negative rates remained substantial (48.1%, 22.2%, 42.3% and 20%), limiting the usefulness of the Amsler grid as a reliable screening tool. These findings are consistent with previous reports describing the limited sensitivity and high false-negative rates of the Amsler test [26, 27]. 

The false-positive rate was 6.6%, indicating that in a small proportion of cases no underlying retinal pathology could be identified despite a positive Amsler test. Possible explanations include an irregular corneal surface, neurological disease, and distortion due to optical aids [17]. 

However, the sensitivity estimates for several retinal pathologies were associated with wide 95% confidence intervals due to the small number of affected eyes, limiting the precision of these estimates. Overall, the findings suggest that the Amsler grid may identify some macular pathologies, but its variable sensitivity and substantial false-negative rates limit its reliability as a standalone screening tool.

### The potential role of telemedicine in the diagnosis of DME

The study suggests that telemedical screening using OCT in combination with a cloud-based patient record system (MedStage^®^) may facilitate earlier detection of DME. Since the examination can be performed by a technician and the ophthalmological report can be made remotely, time and costs may be reduced. This enables screening of a large number of diabetic patients and identification of those requiring treatment in a timely manner. However, the present study was not designed to evaluate the clinical or economic benefits of telemedicine. Within the study framework, the use of the cloud-based platform demonstrated the feasibility of remote ophthalmological assessment.

### Study limitations

This study has several limitations that should be considered when interpreting the results. First, both eyes of each participant were included in the overall study and in several analyses. Although only one eye per patient was included in the primary sensitivity analysis of intrafoveal DME, inter-eye correlation may have affected analyses in which both eyes were retained.

Second, some retinal pathologies and DME subgroups were present in only a small number of eyes, resulting in wide confidence intervals and limiting the reliability of the corresponding effect estimates.

Third, BCVA was not assessed in this study. In addition, information on lens opacity, media clarity, and previous intravitreal treatment (including anti-VEGF therapy) was not available, which limits the interpretation of the relationship between edema area and the subjective perception of metamorphopsia.

Finally, DME was defined based on OCT morphology and quantified using cross-sectional area measurements rather than predefined central retinal thickness thresholds, which may limit comparability with other studies. Furthermore, the DME area was assessed on a single cross-sectional OCT image and therefore represents a two-dimensional measure of a three-dimensional phenomenon, which may not fully reflect the overall extent of intraretinal fluid. Measurement reproducibility was not assessed, as all measurements were performed by a single examiner and no intra- or intergrader agreement analysis was performed.

Future studies should include BCVA and assess its potential influence on the relationship between edema size and Amsler grid performance, while also evaluating the reproducibility of cross-sectional DME area measurements.

## Conclusion

As DR and DME are increasingly prevalent and pose a significant risk to vision, effective screening for early detection and timely treatment is essential. Our results show that the sensitivity of the Amsler grid is insufficient for reliable detection of DME. Very small DME areas (≤ 0.01 mm²) are rarely detected. In addition, the Amsler grid demonstrates limited sensitivity for other maculopathies and is associated with a high false-negative rate. However, abnormal Amsler test results are associated with the presence of retinal pathology and should prompt further ophthalmological evaluation.

Therefore, the Amsler grid may be used as a supplementary home monitoring tool in patients at increased risk of DR or DME, with prompt ophthalmological assessment in case of abnormalities. Nevertheless, regular ophthalmological screening, including OCT-based imaging, remains essential.

Telemedical screening using OCT in combination with a CE-certified digital platform appears feasible and may represent a promising approach for the early detection of DME.

## Data Availability

The datasets used and analysed during the current study are available from the corresponding author upon reasonable request.

## Declarations

### Authors' information

The present work was performed to fulfil the requirements for obtaining the degree of “Dr. med.”.

## Abbreviations

AMD: Age related macular degeneration
BCVA: Best corrected visual acuity
COVID-19: coronavirus disease 2019
CSCR: Central serous chorioretinopathy
CRT: Central retinal thickness
CST: Central subfield thickness
DME: Diabetic macular edema
DR: Diabetic retinopathy
HbA1c: Glycated haemoglobin
HEYEX: Heidelberg Eye Explorer
IDF: International Diabetes Federation
MC: Multicolour
MI: Metamorphopsia index
OCT: Optical coherence tomography
